# Designing and Conducting Healthcare Simulations: Contributions From Social Work

**DOI:** 10.7759/cureus.16193

**Published:** 2021-07-05

**Authors:** Kenta Asakura, Katherine Occhiuto, Sarah Tarshis, Adam Dubrowski

**Affiliations:** 1 School of Social Work, Carleton University, Ottawa, CAN; 2 Faculty of Health Sciences, Ontario Tech University, North Oshawa, CAN

**Keywords:** social work, healthcare simulation, simulation education, competence, meta-competencies

## Abstract

Spurred on by medical education, the last decade has seen a steady increase in simulation-based teaching, learning, and student assessment in social work. Using professional actors trained to portray realistic client scenarios, social work students are afforded risk-free opportunities to rehearse and develop various competencies in working with these simulated patients (SP). This pedagogy is particularly relevant for social work students and practitioners because of the highly vulnerable and marginalized nature of the clients they work with (e.g., suicide intervention, child protection decision-making). In this editorial, we briefly discuss the competency frameworks respectively designed for medicine and other healthcare professionals as well as social work. We highlight ways in which simulation educators might design teaching, learning, and student assessment in preparing healthcare professionals for holistic competence. In doing so, this editorial articulates contributions of social work to broader healthcare simulation education.

## Editorial

Introduction

Working with individuals, families, and communities, social workers collaborate with clients to address presenting concerns through assessment, diagnosis, treatment, and evaluation [1]. They do so in a wide range of settings including hospitals, child protection, mental health organizations, and community non-profit agencies.

Inspired by the pedagogical and outcome-based benefits of simulation in healthcare education, simulation has been increasingly introduced into social work teaching and assessment to prepare social work learners for effective and ethical practice [2]. In social work, simulation "generally refers to a situation where a student or a practitioner engages with a trained actor (i.e., often known as "standardized patient," or SP) or a virtual reality program that portrays a well-designed character and/or practice scenario" [3]. Over the last decade, simulations have been increasingly employed as pedagogical approaches to training students before they begin their field placements [4].

In this editorial, we aim to briefly discuss competency frameworks respectively designed for healthcare (e.g., canMEDS 2015 for medicine) and social work (e.g., holistic competence). Through this discussion, we highlight the contributions social work's holistic and integrated understandings of healthcare can offer to a broader field of simulation-based education.

Social work's holistic competency is defined as "a range of knowledge, values, skills and cognitive and affective processes that include the social workers' critical thinking, affective reactions, and exercise of judgment regarding unique practice situations" [5]. It consists of procedural competence and meta-competence. Procedural competence in social work refers to one's ability to perform observable behaviors, such as interviewing and communication skills (also known as soft skills in other healthcare fields), making a diagnosis, and performing evidence-based treatment (i.e., observable behaviors). Meta-competence, on the other hand, refers to "higher-order, overarching qualities and abilities of a conceptual, interpersonal and personal/professional nature… (including) cognitive, critical, and self-reflective capacities" [6]. Given that social workers specialize in psychosocial support, rather than technical procedurals conducted by other healthcare professionals, meta-competencies are an integral part of their competency framework. This use of meta-competence in social work offers a unique contribution to healthcare. We, therefore, suggest that articulating and incorporating a set of meta-competencies may be beneficial in advancing frameworks of other healthcare professions' education. Specifically, this editorial suggests the following two ways through which social work can contribute to how healthcare simulation educators can design teaching, learning, and student assessment: (1) identify and integrate meta-competencies (i.e., the practitioner's cognitive and affective processes), and (2) incorporate pedagogical activities and assessment measures on meta-competencies.

Competency frameworks

To understand the role of simulation in competence-based education, it is important to ensure a shared understanding of competencies, and competency frameworks. Green and Levy [7] explain that "a competency describes the ability to use a set of related knowledge, skills, and attributes required to successfully perform activities and tasks in a defined setting". They further explain that a competency framework consists of "a combination of well-defined competencies and hierarchical information on how competencies are grouped and connected to work activities, job roles, assessments and more for various applications". Competence-based education, first adopted in medical education, has become a staple of professional education, including social work. The Royal College of Physicians and Surgeons of Canada currently uses CanMEDS as a national competency-based framework for medical education [8]. CanMEDS identifies and describes seven competencies, also referred to as roles: communicator, collaborator, leader, health advocate, scholar, and professional. A physician is considered competent when they can seamlessly integrate these seven competencies into their practice.

**Figure 1:**
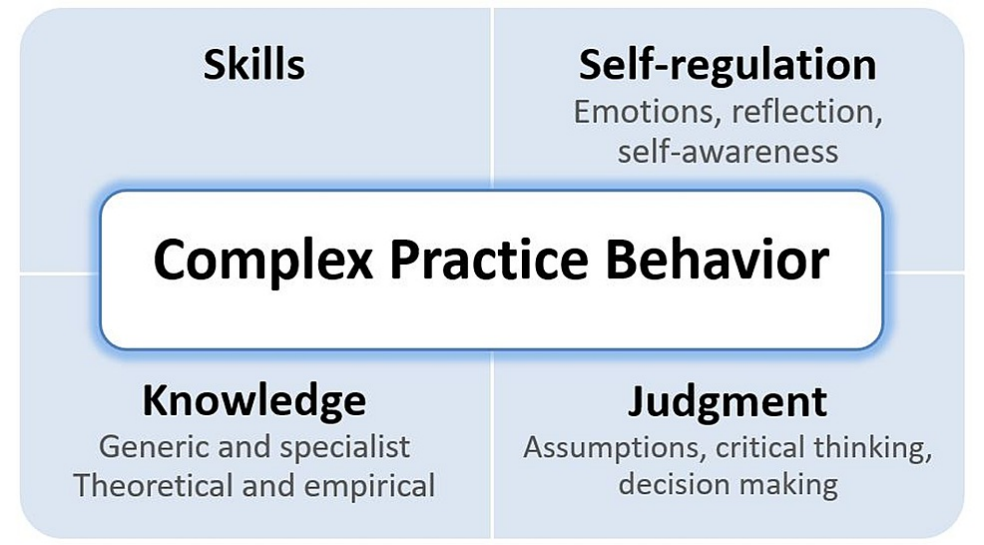
Bogo et al.'s model of holistic competence in social work. Reprinted with permission from the Council on Social Work Education.

Competency frameworks, such as CanMEDS, have contributed significantly to increasing the rigor and relevance of medical education [9]. Critics caution, however, that competence-based education poses some limitations for complex practices such as medicine. Competency frameworks often consist of a list of discrete, directly observable practices that are limited to knowledge and skills within a specific practice context, while medical practice is much more complex than these categorical skill sets [9]. These critics also argue that medical education needs to focus on "higher-order cognitive skills, including analysis, judgment, reflection on previous experience, and 'reading the situation,' to understand a clinical problem and undertake differential diagnosis" [9]. In responding to the critiques of competence-based medical education, Bogo et al. [2] introduced a holistic model of competence for social work that emphasizes more than observable procedural skillsets to also include meta-competencies (Figure 1). While procedural and meta-competencies are defined in two separate categories, these competencies are often intertwined with each other, and competent practice hinges upon both competencies: "How social workers use knowledge, values, and skills is based on the way they bring their own cognitive and affective capacities to bear on interpreting and reacting to the experiences they encounter in practice" [10]. Competent social work practice rests on a dynamic interplay between one's procedural competence and meta-competence.

In social work, meta-competencies go beyond soft skills and refer to higher-order qualities and abilities consisting of cognitive, critical, and self-reflective qualities. A holistic competence framework is needed because social work practice is not an intervention solely dependent upon observable skillsets; rather effective social work practice rests on several contextual factors. Although much of contemporary social work research has focused on evidence-based practice, social work is a professional discipline that has long recognized the contributions of both science and art [11, 12]. While the ability to identify and apply empirically supported interventions (i.e., science) is certainly expected of every social worker, effective practice is also contingent upon the art of practice. This includes non-procedural contextual competencies, such as the worker's critical thinking, reflective capacity, and clinical judgment [10], practice wisdom [13], use of their own lived experience [11], and navigation of a highly dynamic, unique interpersonal process with each client [12]. A robust body of psychotherapy research [14] corroborates this conceptualization of social work practice, in that the type of intervention model or skills used (i.e., procedural competence) predicts patients' successful outcomes far less than other non-procedural factors, such as therapeutic alliance and patient characteristics. The non-technical aspects of practice, such as the practitioner's empathy [15] and managing one's thoughts and feelings towards the client [16] are essential for effective practice. Given that it is social workers' ethical responsibility to serve society's highly vulnerable and marginalized populations (i.e., those impacted by structural inequalities, such as poverty and racism), relying solely on procedural competence is insufficient and in fact, could lead to further harming marginalized clients. This is consistent with the social determinants of health perspective [17], robust empirical evidence suggesting that one's health and wellbeing is highly dependent on their social, economic, and other non-medical factors. As a profession committed to supporting those who are greatly impacted by poverty, racism, and other forms of marginalities, it is essential for social workers to develop the capacity to attend to their assumptions, values, biases, and relational and power dynamics with each client. Social work competence, therefore, emphasizes the process of how one draws from a variety of knowledge, skills, and their own cognitive and affective processes in assessing the client as a whole person and engaging in interventions that work for their unique situations, not just following a particular empirically supported intervention to treat symptoms.

In healthcare education (e.g., pharmacy, nursing), there have been some promising expansions of competency frameworks. These include attending to the more complex elements of practice by integrating higher-order cognitive skills, such as the practitioner's empathy [18], emotional intelligence [19, 20], and reflection [21]. The inclusion of these higher-order skills as a part of competencies is a welcoming addition. This work, however, remains peripheral, and there remains a need to further articulate and integrate such higher-order competencies [20]. 

Simulation-based social work education

Like medicine and other healthcare professional education, simulation has become a highly valued, common experiential pedagogy in social work because of its capacity to "bridge the learning in the classrooms and the field" [2]. In social work education, simulation is primarily used in two ways: (1) teaching and learning in the classroom and (2) student assessment.

Simulation-Based Teaching and Learning

Simulation is used in social work teaching in varying formats that range from highly standardized [2] to improvisational [22]. A wide array of formats is employed including face-to-face with a live SP [2], video-recorded web-based SP [23], and virtual or artificial intelligence (AI)-based [24, 25, 26]. Our team has argued that simulation is not a stand-alone teaching tool, instead, it is how educators engage simulation that makes it a meaningful learning instrument for students [24]. Meaningful learning requires carefully scaffolding students' learning in preparation for the simulation activities. Informed by Vygotsky's instructional scaffolding framework [27], students, for instance, practice their interviewing skills on a virtual simulation platform to focus on procedural competencies, before engaging with a live-SP, which requires students to incorporate meta-competencies, such as self-regulation and judgment, into practice. 

With so much of social work practice being contextual, the development of realistic and complex scenarios for simulation is imperative. Case scenarios must be designed to capture the dynamic interplay across the intrapsychic (e.g., thought processes, emotional states), interpersonal (e.g., relational patterns), social (e.g., identities, school/work), and structural (e.g., historically-rooted marginalization, privileges) domains. To ensure that scenarios are developed without perpetuating stereotyping, appropriating, or oversimplifying the complex experiences of the marginalized people we work with, we consult with community partners (e.g., agencies, practitioners) in designing case studies.

Much of the learning around meta-competencies in social work learning requires designing spaces for students to reflect on their engagements with simulation, and to learn vicariously through their peers' work [28]. Our team provides ample opportunities for students to engage in reflective class discussions about not only procedural competencies (e.g., skills used with SPs) but also meta-competencies. Instructors, for instance, might pose a reflective question to evoke the students' thoughts and feelings about the simulated practice, such as "What did you notice internally (i.e., emotional and somatic reactions) as you sat with this client's loss of her mother? Where do you think that these reactions came from?" In addition to class discussions, this type of reflection about one's meta-competencies happens in other pedagogical forms, such as debriefing with peers, course-based assignments, guided observation of their peers' simulations, and observing and reflecting on the feedback provided to colleagues. Through this balance of attention to procedural and meta-competencies, we aim to prepare social work students for holistic practice. 

Simulation-Based Student Assessment:

In assessing student learning outcomes, Bogo and colleagues [2] expanded on healthcare education to develop a social work version of an Objective Structured Clinical Examination (OSCE) to assess both dimensions of competence: procedural competencies (e.g., developing a collaborative relationship, utilizing specific interviewing skills, assessment) and meta-competencies (e.g. ability to engage in critical thinking, demonstrates self-awareness). OSCE adapted for Social Work (OSCE-SW) consists of two components. The first component is a timed simulation with an SP. Students are expected to demonstrate various competencies that are rated by an examiner via a standardized scale. At the end of the simulation, a short period of time is provided for the student to receive oral feedback from the examiner and the SP.

The second component requires students to independently respond to a series of reflective questions designed to assess students' meta-competence for social work practice, such as emotional regulation, judgment, critical thinking, and the students' ability to reflect on their feelings, thoughts, and responses to the client. The students produce a written reflection describing their conceptual understanding of the client, internal decision-making processes, their understanding of the feedback they received, as well as goals for future professional growth. The examiner rates the written reflections via a standardized form, evaluating the students' ability to engage with meta-competencies.

Discussion

One major critique of the medical competency framework is its heavy reliance on procedural competencies. This is not surprising or always problematic given that historically simulation was focused on the very technical nature of medicine and other healthcare practices situated in a biomedical culture. In recognizing the complex and nuanced needs of health and social service users, social work has proposed and embraced a more holistic and integrated competency framework that includes meta-competence [10]. Leveraging social work's holistic and integrative competence framework, we suggest two ways that healthcare educators can attend to non-procedural aspects of professional competencies in designing and conducting simulation education: 1) identify and integrate meta-competencies in designing simulations, and 2) incorporate pedagogical activities and assessment measures on meta-competencies.

1. Identify and Integrate Meta-Competencies in Designing Simulations

Building on the existing body of work that critiques medical competency frameworks [9], there is no doubt that practitioners' non-procedural competencies, such as critical thinking, reflective capacity, values and ethics, and clinical judgment, can further strengthen healthcare practice. With growing evidence of social determinants of health [17], healthcare educators might greatly benefit from identifying essential meta-competencies for their respective professions.

Once key meta-competencies are identified, healthcare educators can design simulations that allow students to experientially practice and demonstrate these meta-competencies. For instance, attending to diversity and difference is one area of meta-competence required of social workers. In the context of social work practice, this means that social workers are expected to critically examine both the client’s and their own social identities (e.g., race, gender) and associated social locations (e.g., privileges, experiences of social marginalization). A competent social worker is then able to make a professional judgment in approaching the client by accounting for power dynamics within the therapeutic relationship. In medicine, simulation educators could design case scenarios that incorporate or focus on targeted meta-competencies. This can support students in focusing on more holistic approaches to practice and can lead to more effective healthcare delivery.

2. Incorporate Pedagogical Activities and Assessment Measures on Meta-Competencies 

Once simulations are designed to allow students to practice identified meta-competencies, simulation educators can incorporate relevant pedagogical activities. When engaging in simulation-based teaching in social work, for instance, time is set aside for students to reflect on their work with SPs. Further, students are guided by educators to pay attention to their emotional responses to SPs and to engage in critical discussions around their reflections with their colleagues. Students might also be required to look back on specific practice examples from their field education, and to reflect on how factors such as emotional regulation, power, and culture impacted the client encounter. By training students to explicitly focus on these meta-competencies, social workers are trained to make informed clinical judgments for assessment and treatment based not only on presenting issues, but also on the many contextual factors involved.

Similarly, when using OSCE for student assessment, simulation educators can incorporate assessment measures relevant to identified meta-competencies. Unlike in most other OSCE formats, in the OSCE-SW, students are required to complete a written reflection focused on their meta-competencies immediately following the feedback students receive from the examiner after engaging with the SPs. The reflective component consists of a series of open-ended questions that focus on how students perceive their performance during the simulation, as well as students’ understanding of the feedback they received. This reflective exercise, which is a component art of formative assessments, is designed to measure students’ cognitive processes of how they link theory and evidence to practice; the students' affective processes of how they engage their own emotions in working with SPs; and how students integrate feedback into their learning. Assessment measures like this type of reflective assessment offer educators and students a method to direct greater attention to meta-competencies that can promote a more integrated, holistic healthcare. Further, they provide opportunities for educators to work with students on how they integrate feedback into their practice, which supports continued learning and growth post-graduation.

Conclusion

The use of simulation in medical and other healthcare education has laid strong foundations for its use in a wide range of professional training programs including social work. A spotlight on social work’s holistic framework of competency, which integrates procedural and meta-competencies, demonstrates the value of interprofessional dialogue around the different ways simulation is being employed. Further interprofessional dialogue and collaboration have the potential to further develop the use of simulation in training competent healthcare professionals. 
